# Inorganic Nanomedicine—Mediated Ferroptosis: A Synergistic Approach to Combined Cancer Therapies and Immunotherapy

**DOI:** 10.3390/cancers16183210

**Published:** 2024-09-20

**Authors:** Adityanarayan Mohapatra, Ayeskanta Mohanty, In-Kyu Park

**Affiliations:** 1 Department of Biomedical Sciences and BioMedical Sciences Graduate Program (BMSGP), Chonnam National University Medical School, Gwangju 61469, Republic of Korea; mnaditya95@gmail.com (A.M.); ayeskanta99@gmail.com (A.M.); 2 DR Cure Inc., Hwasun 58128, Republic of Korea

**Keywords:** ferroptosis, inorganic nanomedicine, immunotherapy, radiotherapy, chemotherapy, phototherapy, combined cancer therapy

## Abstract

**Simple Summary:**

Ferroptosis is a regulated form of cell death driven by iron and lipid peroxidation, showing great potential in cancer therapy, particularly for tumors that resist conventional treatments. In our review, we focus on the role of inorganic nanoparticles in inducing ferroptosis and how this can be combined with therapies such as chemotherapy, radiotherapy, immunotherapy, and phototherapy. These nanoparticles not only trigger ferroptosis to kill cancer cells but also enhance the body’s immune system response against tumors. The review discusses significant progress in using nanomedicine to promote ferroptosis and highlights its potential to improve treatment outcomes. Additionally, we explore the metabolic processes involved, particularly how interactions between iron, lipid, and redox pathways regulate ferroptosis in tumor cells. Understanding these mechanisms is crucial for developing more effective cancer treatments. As research in this field advances, the opportunity to move ferroptosis-based therapies from the lab to clinical practice increases. This combination of ferroptosis with inorganic nanomedicine offers a promising strategy for creating more targeted and powerful cancer therapies, ultimately improving patient survival and expanding treatment possibilities.

**Abstract:**

Ferroptosis, a form of regulated cell death characterized by iron-dependent lipid peroxidation, has generated substantial interest in cancer therapy. Various methods have been developed to induce ferroptosis in tumor cells, including approved drugs, experimental compounds, and nanomedicine formulations. Unlike apoptosis, ferroptosis presents unique molecular and cellular features, representing a promising approach for cancers resistant to conventional treatments. Recent research indicates a strong link between ferroptosis and the tumor immune microenvironment, suggesting the potential of ferroptosis to trigger robust antitumor immune responses. Multiple cellular metabolic pathways control ferroptosis, including iron, lipid, and redox metabolism. Thus, understanding the interaction between tumor metabolism and ferroptosis is crucial for developing effective anticancer therapies. This review provides an in-depth discussion on combining inorganic nanoparticles with cancer therapies such as phototherapy, chemotherapy, radiotherapy, and immunotherapy, and the role of ferroptosis in these combination treatments. Furthermore, this paper explores the future of tumor treatment using nanomedicine, focusing on how inorganic nanoparticles can enhance ferroptosis in tumor cells and boost antitumor immunity. The goal is to advance ferroptosis-based nanomedicine from the laboratory to clinical applications.

## 1. Introduction

Cancer, one of the world’s leading causes of death, remains challenging to treat because of its complex and adaptable nature. Researchers have uncovered various cellular processes in the quest for new cancer therapies, with ferroptosis emerging as a promising target. In 2012, Brent R. Stockwell defined ferroptosis as a unique type of regulated cell death reliant on iron and induced by lipid reactive oxygen species (L-ROS) [1]. Unlike other regulated cell death forms such as apoptosis, cuproptosis, necroptosis, and pyroptosis, ferroptosis is distinguished by its specific cellular morphology, biochemistry, and genetic characteristics. Since its discovery, ferroptosis has attracted substantial interest due to its unique mechanisms and potential to bypass resistance to traditional cancer treatments.

**Figure 1:**
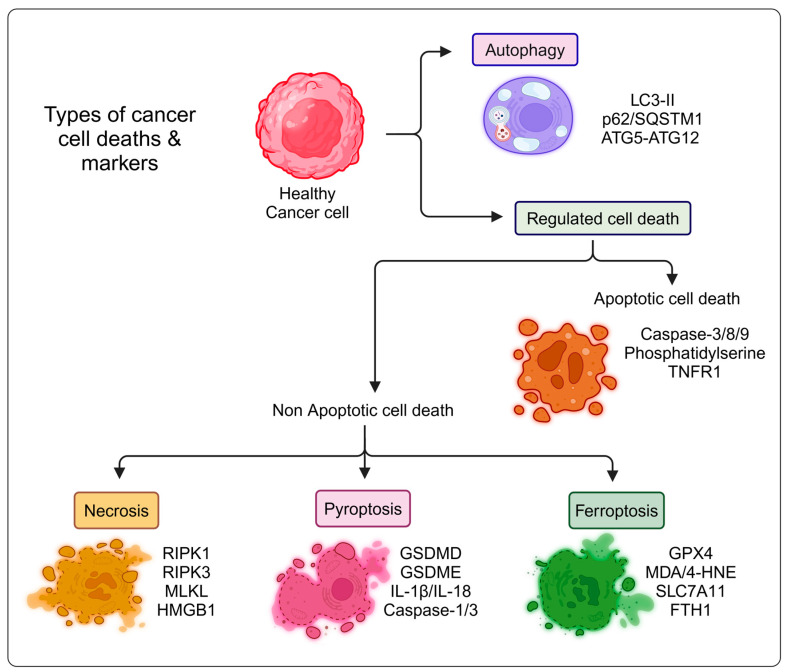
Schematic illustration of cancer cell death types and markers. This figure was created with BioRender.com.

Ferroptosis is a distinct form of regulated cell death, characterized by iron-dependent lipid peroxidation, which differentiates it from apoptosis, necrosis, and pyroptosis [2,3]. Unlike apoptosis, which involves caspase activation, DNA fragmentation, and phosphatidylserine externalization (detected by Annexin V), ferroptosis does not rely on these mechanisms. Instead, it is marked by the depletion of cellular antioxidants, particularly glutathione (GSH), the inactivation of glutathione peroxidase 4 (GPX4), and the accumulation of intracellular iron and lipid peroxides (Figure 1) [4,5]. Morphologically, ferroptotic cells display mitochondrial shrinkage and increased mitochondrial membrane density without the nuclear fragmentation or plasma membrane blebbing seen in apoptosis. In contrast to necrosis, which is an unregulated process involving membrane rupture and inflammation, ferroptosis is a tightly regulated mechanism that causes oxidative damage without inducing an inflammatory response [4,6]. The susceptibility of cells to ferroptosis, as opposed to apoptosis or necrosis, is heavily influenced by cell type and specific regulatory mechanisms. This depends on the expression of key ferroptosis regulators like GPX4, SLC7A11, and ferritin, which govern a cell’s ability to manage oxidative stress [6]. For example, cancer cells with altered iron metabolism and higher ROS production are more prone to ferroptosis, while cells with stronger antioxidant defenses, such as those expressing high levels of GPX4, are more resistant [7]. Neurons, due to their high oxidative demands, may be more vulnerable to ferroptosis under oxidative stress conditions, whereas immune cells may be more inclined toward apoptosis [7,8,9]. Thus, the decision for a cell to undergo ferroptosis, apoptosis, or necrosis is context-dependent, influenced by the cell’s ability to handle iron, manage oxidative stress, and respond to specific signaling pathways. In cancer cells, ferroptosis is tightly regulated by pathways linked to iron metabolism and lipid peroxidation. Intracellular iron catalyzes ROS production via Fenton chemistry, and the resulting imbalance between ROS generation and antioxidant defenses, particularly reduced GPX4 activity, leads to ferroptotic cell death. Mitochondria are the primary source of intracellular ROS although the endoplasmic reticulum and NADPH oxidase also contribute. Normally, antioxidant defenses like GPX4, heat shock factor-binding protein 1 (HSBP1), and nuclear factor erythroid 2-related factor 2 (Nrf2) suppress ferroptosis by reducing ROS levels and limiting iron uptake [10]. Conversely, proteins like P53 and NADPH oxidase promote ferroptosis by increasing ROS production. Since the discovery of GPX4-centered ferroptosis mechanisms in 2014, additional pathways, both GPX4-dependent and independent, have been identified, offering a broader understanding of ferroptosis regulation and its therapeutic potential in cancer [11]. Thus, ferroptosis emerges as a unique and specialized form of cell death that hinges on oxidative stress and iron metabolism, with significant implications for cancer therapy.

**Figure 2:**
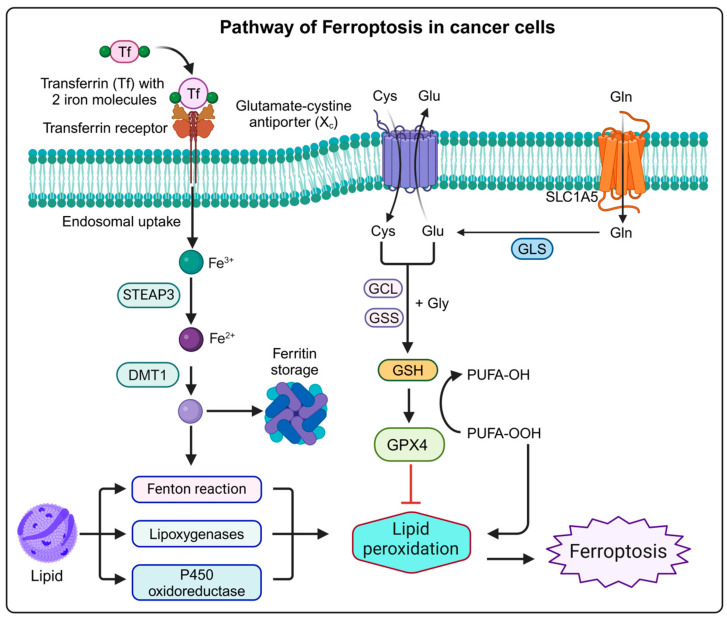
Schematic illustration of the ferroptosis pathway mechanism. This figure was created with BioRender.com.

Ferroptosis is driven by a complex interplay of iron accumulation, ROS production, and lipid peroxidation [3]. The process begins with the buildup of intracellular iron, which facilitates the Fenton reaction, generating highly reactive ROS that induces lipid peroxidation, particularly targeting polyunsaturated fatty acids (PUFAs) on cell membranes (Figure 2). This peroxidation disrupts membrane integrity and contributes to cell death. Mitochondria further exacerbates ROS production through oxidative phosphorylation, increasing oxidative stress and lipid damage. Ferroptosis is characterized by specific morphological features, such as mitochondrial atrophy and increased membrane density. In contrast to ferroptosis, apoptosis and oxytosis are other forms of cell death that can also be triggered by ROS but differ significantly in their mechanisms. Apoptosis, though often initiated by ROS, involves a cascade of intracellular events that lead to cell shrinkage, chromatin condensation, and the formation of apoptotic bodies. It is typically associated with caspase activation and results in the controlled, noninflammatory removal of cells. On the other hand, oxytosis, or oxidative cell death, is driven by excessive oxidative stress causing damage to various cellular components like lipids, proteins, and DNA [12]. Oxytosis can lead to cell death through severe oxidative damage to various cellular components, including proteins and nucleic acids, without necessarily involving the iron-dependent lipid peroxidation pathway [12]. Unlike ferroptosis, which is specifically associated with iron-mediated lipid peroxidation, oxytosis encompasses a broader range of oxidative damage and lacks the distinct metabolic and morphological changes characteristic of ferroptosis. Thus, while ROS can trigger both apoptosis and oxytosis, ferroptosis is uniquely defined by its reliance on iron-driven lipid peroxidation and specific cellular changes. The regulation of ferroptosis is intricately linked to disruptions in iron metabolism, lipid metabolism, and amino acid metabolism. Iron metabolism encompasses absorption, utilization, recycling, and storage [13]. Disruptions in these processes can lead to excessive intracellular iron, triggering the Fenton reaction and producing ROS that contribute to oxidative stress and ferroptosis. Iron also plays essential roles in DNA and ATP production and is a key component of the mitochondrial electron transport chain. Imbalances in iron uptake or storage mechanisms, such as those involving transferrin, ferritin, and divalent metal transporters, can lead to iron overload, exacerbating ROS production and promoting ferroptosis [14]. Lipids are crucial for various cellular functions, including energy storage, membrane formation, and signal transduction [15]. Disruptions in lipid metabolism, particularly concerning fatty acid composition, can drive ferroptosis. PUFAs are highly susceptible to oxidative damage by ROS, leading to lipid peroxidation—a hallmark of ferroptosis. Enzymes such as acyl-CoA synthetase long-chain family member 4 (ACSL4) promote PUFA production, thereby facilitating ferroptosis [16]. In contrast, monounsaturated fatty acids (MUFAs) can mitigate ferroptosis by reducing membrane lipid susceptibility to oxidation, underscoring the impact of lipid composition on cell death. Amino acid metabolism, particularly involving GSH, is also crucial in regulating ferroptosis. GSH acts as a key antioxidant that neutralizes ROS and prevents oxidative damage [17]. Disruptions in amino acid metabolism affect GSH levels, influencing ferroptosis. The system XC—complex, which imports cystine for GSH synthesis, and GPX4, which utilizes GSH to reduce lipid peroxides, are central to this regulation. Impairments in system XC— or reductions in intracellular cysteine levels lead to decreased GSH production and inadequate ROS detoxification, thus promoting ferroptosis [17].

The potential of ferroptosis in cancer therapy, coupled with advancements in nanotechnology, presents numerous opportunities and challenges for their integration into cancer treatment. Nanoparticle-based delivery of small-molecule ferroptosis inducers primarily focuses on system Xc⁻ inhibitors (e.g., erastins, sulfasalazine, sorafenib) and GPX4 inhibitors (such as RSL3 and altretamine), as well as approaches inducing GPX4 degradation (e.g., FIN56) and GSH depletion (e.g., buthionine sulfoximine, DPI2), offering potential therapeutic avenues for cancer [18]. For instance, Zhao et al. developed a hypoxia-responsive polymer micelle encapsulating RSL3, facilitating its release in the tumor microenvironment under hypoxic conditions, thereby inhibiting GPX4 activity and inducing ferroptosis [19]. While small-molecule delivery via organic nanoparticles has shown potential, it is less effective at triggering ferroptosis compared with inorganic nanoparticles (NPs). Inorganic NPs, such as iron oxide (Fe), manganese (Mn), copper (Cu), zinc (Zn), and platinum (Pt) present distinct advantages in cancer therapies, particularly in ferroptosis induction, due to their superior chemical stability, enhanced surface modification properties, and tunable physical properties, including size, shape, magnetism, and optical responsiveness. These properties enable them to more effectively target tumor cells and induce ferroptosis. A major advantage of inorganic NPs lies in their ability to catalyze ROS generation through Fenton-like reactions, a critical mechanism for inducing ferroptosis by disrupting redox homeostasis in cancer cells. Conversely, organic NPs lack intrinsic catalytic activity and often require additional modifications to achieve similar levels of ROS generation, making inorganic NPs more efficient in ferroptosis induction. Furthermore, inorganic NPs allow for precise control over ROS production, enhancing selectivity toward cancer cells while minimizing collateral damage to healthy tissues [20]. Organic NPs often face challenges in achieving such precision due to their unpredictable degradation and less stable drug release profiles in biological environments. Inorganic NPs, with their enhanced stability, offer more predictable and sustained therapeutic effects. Additionally, their ability to be functionalized with targeting ligands further enhances therapeutic precision, whereas organic NPs, with their complex structures, may struggle to achieve similar precision. Moreover, the intrinsic magnetic and optical properties of inorganic NPs enable real-time imaging and monitoring, making them well-suited for theranostic applications that integrate diagnosis and treatment. In contrast, organic NPs typically require complex modifications to achieve such dual functionality, limiting their versatility in clinical settings. Overall, the superior stability, catalytic activity, precision targeting, and multifunctionality of inorganic NPs make them a more robust and effective option compared with organic NPs, particularly in overcoming tumor resistance mechanisms and enhancing the efficacy of ferroptosis-based cancer therapies [20].

Given the rapid advancements in ferroptosis research, uncovering this process’s intricate mechanisms is crucial. Therefore, this review focuses on exploring the milestones and molecular processes of ferroptosis, particularly when using inorganic NPs to trigger it, and modulating immune responses in cancer, especially in combination therapies.

## 2. Inorganic Nanoparticles Induce Ferroptosis-Mediated Cell Death

**Figure 3:**
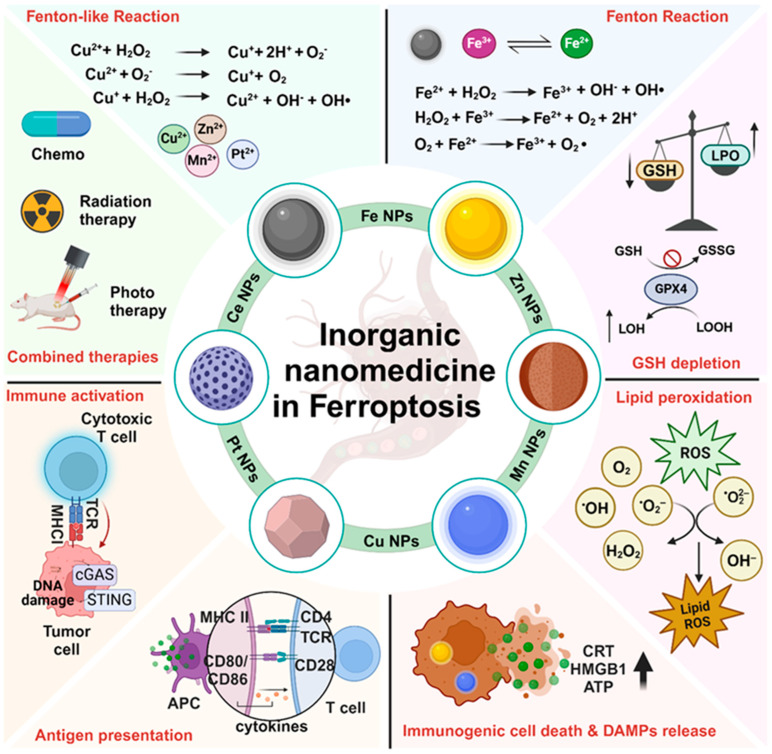
Schematic illustration of the ferroptosis mechanism mediated by inorganic nanomedicine and its application in integrated cancer therapies. This figure was created with BioRender.com.

In recent years, scientists have investigated how different types of inorganic NPs can be used to fight cancer by triggering ferroptosis. This process involves the buildup of iron and lipid peroxides inside cancer cells, destroying them while sparing healthy cells. Inorganic NPs have gained significant attention as innovative tools for inducing ferroptosis, which selectively targets cancer cells while sparing healthy tissues. Various metal-based NPs, including iron, copper, manganese, zinc, and platinum, play distinct roles in this process. Fe-based NPs are key to catalyzing the Fenton reaction, generating ROS that drive lipid peroxidation, a hallmark of ferroptosis. Cu-based NPs similarly promote ROS production via redox cycling, increasing oxidative stress within cancer cells. Mn-based NPs boost ROS levels further, accelerating lipid peroxidation, while Zn-based NPs interfere with cellular antioxidant defenses by depleting GSH, thus promoting the ferroptosis pathway. Pt-based NPs disrupt the redox balance, enhancing lipid peroxidation, especially when combined with oxidative stress-inducing treatments. These nanoparticles are uniquely designed to be precisely targeted to cancer cells, reducing off-target toxicity to healthy tissues. Moreover, when used in combination with therapies such as photothermal therapy (PTT) or photodynamic therapy (PDT), these NPs not only intensify ferroptosis but also modulate the immune response, enhancing the overall antitumor effect and providing a promising dual strategy for advancing cancer treatment. These NPs work by adjusting iron levels and influencing how lipids are processed in cancer cells, ultimately causing them to undergo ferroptosis [21]. The unique characteristics of these NPs allow them to be precisely targeted to cancer cells, minimizing harm to healthy tissues. Furthermore, when combined with other therapies, such as PTT and PDT, these NPs help modulate the immune response against cancer cells (Figure 3). This dual approach holds great promise for advancing cancer treatment.

### 2.1. Iron Oxide NP-Based Ferroptosis

Iron oxide NPs have become increasingly valuable in biomedicine because of their distinctive characteristics and size. These NPs have proven effective in biomedical applications such as delivering drugs directly to targeted areas, enhancing magnetic resonance imaging (MRI) for better diagnostics, and even in hyperthermia therapy to selectively target and destroy cancer cells [22]. Besides their applications in drug delivery and imaging, iron oxide NPs can induce ferroptosis-mediated cell death, which is beneficial for cancer therapy. In acidic environments, iron oxide NPs degrade and release Fe^+^ ions. These ions can interact with hydrogen peroxide (H_2_O_2_) through the Fenton reaction to generate ROS (see below) [23]. This reaction involves the conversion between ferric (Fe^3+^) and ferrous (Fe^2+^) forms of iron, oxidizing saturated fatty acids in cell membranes [24].

Fe-based Fenton reaction: Fe^2+^ + H_2_O_2_ → Fe^3+^ + OH^−^ + OH

These biochemical changes contribute to the DNA damage and destruction of tumor cells, representing a potential pathway for iron oxide-mediated cancer treatment.

In a recent study, researchers developed a nanotheranostic system using a modified oxaliplatin prodrug and poly(ethylene) glycol (PEG) on Fe (III)-porphyrin metal–organic frameworks (PCN-Oxpt/PEG). These frameworks had MRI and fluorescence capabilities, enhanced by the PEG coating, which improved their biological half-life. The nanoplatform demonstrated a novel approach to inducing ferroptosis through several mechanisms: (i) the prodrug depleted GSH, hindering GPX4 activation; (ii) oxaliplatin stimulated CD8+ T cells and produced IFN-γ, further inhibiting GPX4; (iii) internalized oxaliplatin-triggered H_2_O_2_ generation via NADPH oxidases (NOXs) and superoxide dismutase-mediated superoxide anion (O^2⋅−^) dismutation, leading to lipid peroxidation. This triple-action strategy combining chemotherapy, ferroptosis induction, and immunotherapy showed promising results in treating 4T1 tumor-bearing mice, outperforming single therapies [25]. Similarly, Yu et al. developed a self-assembled nanoparticle combining a Fe^2+^-modified mesoporous zeolitic imidazolate framework-8 with tannic acid (TA) and cationic chlorin e6-poly(amidoamine) (Ce6-PAMAM) for treating non-small-cell lung cancer. When exposed to a near-infrared (NIR) laser, the nanoparticle generated cytotoxic ROS such as singlet oxygen (^1^O_2_) and hydroxyl radicals (⋅OH) through Ce6-induced PDT and Fe^2+^-mediated Fenton reactions [26]. The controlled release of TA ensured sustained Fenton chemistry, highlighting the potential of this approach for cancer treatment. Overall, these multifunctional nanoplatforms with imaging and responsive therapy capabilities represent a promising advancement in overcoming the limitations of conventional cancer treatments.

### 2.2. Copper NP-Based Ferroptosis

Copper is essential for numerous enzymes, including mitochondrially encoded cytochrome c oxidase I (MT-CO1) and superoxide dismutase (SOD1), which play key roles in the electron transport chain and the antioxidant system in mammalian cells. Both copper deficiency and excess can disrupt normal cellular functions and lead to cell death. Hence, copper-based strategies for inducing ferroptosis represent a promising avenue in cancer treatment. Copper NPs facilitate ROS generation through redox cycling between Cu (I) and Cu (II) states, similar to the iron-dependent Fenton reaction. Furthermore, these copper ions deplete GSH by converting it to its oxidized form. This, in turn, impairs the function of GPX4, a critical enzyme for preventing lipid peroxidation. The decrease in GSH levels and GPX4 activity results in the buildup of lipid peroxides, leading to the oxidative damage of cell membranes and inducing ferroptosis. Song and colleagues developed self-assembled copper-alanine nanoparticles (CACG) loaded with cinnamaldehyde (Cin) and glucose oxidase (GOx) to enhance ferroptosis and immunotherapy. CACG effectively delivered Cu^2+^, Cin, and Gox to the GSH-rich and acidic tumor microenvironment (TME). Cin depleted GSH via Michael addition and reduced Cu^2+^ to Cu^+^ for further GSH depletion. The combined Cu^+^-catalyzed Fenton reactions and GOx-catalyzed reactions ensured continuous ROS generation, overcoming H_2_O_2_ limitations in the TME. This efficient GSH depletion and ROS production enhanced ferroptosis in vivo [27]. On the other hand, Cu_2_O NPs were synthesized and subsequently coated with gold (Au) NPs to create Cu_2_O@Au NPs to enhance ferroptosis. Once delivered into cancer cells, the released Cu_2_O consumed endogenous H_2_S within colorectal cancer cells, forming Cu_31_S_16_ NPs. The consumption of H_2_S further enhanced the therapeutic effect against colorectal cancer. Moreover, the activation of the Cu^2+^/Cu^+^ catalytic cycle further depleted GSH, resulting in the buildup of lipid peroxides. This synergistic approach, combining CDT/starvation therapy with Cu_2_O@Au nanocomposites, may enhance ferroptosis and elicit a strong immune response against tumors [28]. However, it is important to distinguish copper-based ferroptosis from another copper-induced form of cell death called cuproptosis. Cuproptosis is distinct from ferroptosis as it involves the accumulation of copper within mitochondria, leading to the aggregation of mitochondrial proteins and the destabilization of iron-sulfur cluster proteins, which eventually causes cell death through a proteotoxic stress mechanism. Unlike ferroptosis, which is dependent on lipid peroxidation, cuproptosis is driven by copper’s direct interference with mitochondrial respiration. The copper-based nanoparticles mentioned above trigger ferroptosis, not cuproptosis, because their primary mechanism involves ROS generation through redox cycling and the depletion of GSH, leading to lipid peroxidation and the inhibition of GPX4. This lipid peroxidation-driven process is characteristic of ferroptosis, whereas cuproptosis does not involve these pathways but instead focuses on mitochondrial protein aggregation and metabolic dysfunction.

Exogenous copper binds directly to GPX4 protein cysteines (C107 and C148), causing GPX4 to form aggregates and be tagged for degradation [29]. Tax1 binding protein 1 (TAX1BP1) is necessary for copper-mediated ferroptosis, acting as an autophagic receptor for GPX4 breakdown. A study reported that copper enhanced the antitumor activity of ferroptosis inducers in a mouse model of pancreatic cancer [29]. Moreover, copper-containing NPs proved effective in delivering copper to induce ferroptosis in colorectal cancer cells [30]. Therefore, ferroptosis is a metal-dependent mode of cell death that involves both iron and copper.

### 2.3. Manganese NP-Based Ferroptosis

Manganese catalyzes Fenton-like reactions and produces higher levels of ROS than iron, making it a valuable element for biological processes because of its biocompatibility [31]. Manganese, known for its multivalence and high-spin properties, acts as a key cofactor in metalloenzymes that influence cell metabolism and the redox balance [32]. Although limited research has connected ferroptosis to manganese ions alone, Mn^2+^ ions released from MnO_2_ nanoparticles were proven to convert endogenous H_2_O_2_ into toxic •OH via Fenton-like reactions. This discovery led to the integration of Mn^2+^ into iron-based nanomaterials to create manganese carbonate-deposited iron oxide (FMO) nanoparticles to enhance ferroptosis. FMO is stable under physiological conditions but releases Mn^2+^ and Fe^2+^/Fe^3+^ ions in the acidic tumor environment. Ferroptosis relies on iron and is linked to the Fenton reaction; thus, combining manganese and iron ions enhances cell ferroptosis because iron induces the process and manganese boosts the Fenton reaction. A study combined a cisplatin prodrug (Pt (IV)) with FMO to create a Pt-FMO nanoplatform [33]. Activated by intracellular reducing agents like GSH, Pt (IV) was converted into potent cisplatin within tumor cells, lowering the levels of GSH, which typically scavenges ROS. Therefore, the Pt-FMO NPs initiated cascade reactions using the Pt drug to elevate intracellular H_2_O_2_ levels. This H_2_O_2_ was then converted into highly toxic •OH through the Fenton reaction, catalyzed by manganese/iron ions released from FMO, promoting ferroptosis combined with an apoptotic promoter in cancer cells [33].

In the TME, characterized by hypoxia and high GSH levels, lipid peroxide (LPO) production is inhibited and GPX4 actively clears LPO, limiting the effectiveness of ferroptosis therapy. To overcome these challenges, Mengyao et al. introduced a new triple-enhanced ferroptosis amplifier named Zal@HM-PTBC, composed of hollow manganese nanoparticles (HM-NPs), which facilitated the loading of the drug zalcitabine (ZAL) because of their porous structure. These NPs were subsequently modified with bovine serum albumin-copper sulfide NPs (BSA-CuS) and triphenylphosphine (TPP). TPP incorporation ensured the mitochondrial targeting of the Zal@HM-PTBC complex. Interestingly, the degradation of Zal@HM-PTBC within the TME depleted GSH and reduced both GPX4 activity and LPO clearance. This process also induced the targeted release of ZAL and fostered autophagy-dependent LPO accumulation. Moreover, the photothermal conversion of BSA-CuS under 808 nm laser irradiation not only enabled PTT but also enhanced Mn^2+^-mediated Fenton-like reactions, increasing ROS production. This comprehensive strategy amplified LPO accumulation and promoted ferroptosis for pancreatic cancer treatment [34].

### 2.4. Zinc NP-Based Ferroptosis

Zinc oxide nanoparticles (ZnO NPs) are widely used in areas such as food additives for their nutritional benefits, sunscreen for UV protection, and antimicrobial agents for skin defense [35]. Additionally, ZnO NPs are important in medical applications, including drug delivery, tissue regeneration, and bioimaging [36]. However, growing evidence reveals the toxic effects of ZnO NPs on animals, particularly impacting the respiratory, cardiovascular, and nervous systems [37,38,39]. These toxic effects are mainly due to oxidative stress, inflammation, and cell death. ZnO NPs can induce various forms of cell death, such as apoptosis, necrosis, and autophagy, in both cultured cells and animal models. Recent research has also shown that ZnO NPs interact with intracellular iron, significantly increasing the production of lipid peroxidation products like malondialdehyde (MDA), which are associated with ferroptosis, an iron-dependent form of cell death [40]. Wang et al. developed ZnO nanoparticles to investigate their catalase-mimicking activities in combination with ferroptosis and photodynamic therapy [41]. They modified ZnO NPs with the photosensitizer Ce6 to create zinc oxide–chlorin e6 nanoparticles (ZnO-Ce6 NPs). Upon treating cancer cells with both ZnO NPs and ZnO-Ce6 NPs, a significant increase in lipid peroxidation levels was observed. Additionally, a key marker of ferroptosis, GSH depletion, was noted in cells treated with ZnO NPs, along with a reduction in GPX4 protein levels [41]. These findings suggest that ZnO NPs effectively induce ferroptotic cell death during anticancer therapy.

### 2.5. Platinum NP-Based Ferroptosis

Pt-based compounds are known for their ability to induce DNA damage and disrupt redox homeostasis due to their strong affinity for thiol-rich biomolecules such as GSH [42]. The Pt-based chemotherapeutic agent cisplatin has been recently documented to elevate intracellular lipid ROS accumulation at higher doses over prolonged exposure, leading to ferroptotic cell death [43,44]. Huang et al. have engineered gold nanostars coated with platinum (Pt-AuNS) to overcome cancer drug resistance by combining photothermal therapy with ferroptotic therapy [45]. Upon NIR laser irradiation, these nanostructures release Pt ions, leading to GSH depletion and inactivation of GPX4. In TRAMP-C1 cancer cells, Pt-AuNS treatment resulted in a time-dependent increase in Pt release, with a Pt concentration of 25 µM causing cytotoxicity through lipid peroxidation and ROS generation [45]. The therapeutic efficacy of irradiated Pt-AuNS was assessed in a drug-resistant xenograft model. To confirm ferroptosis involvement in tumor eradication, mice treated with Pt-AuNS received daily intraperitoneal injections of liproxstatin-1, a ferroptosis inhibitor, over a 10-day period. Administration of liproxstatin-1 compromised the therapeutic efficacy of Pt-AuNS, facilitating tumor progression in the treated mice [45].

## 3. Inorganic Nanoparticle-Based Ferroptosis in Combinational Anticancer Therapy

### 3.1. Ferroptosis and Cancer Immunotherapies

Cancer immunotherapy is a promising approach that uses the patient’s immune system to fight cancer. This method takes advantage of tumor-specific antigens found on cancer cells. These antigens can interact with immune components like antibodies or T cell receptors, leading to an immune response against the tumor. Researchers have explored various ways to improve cancer immunotherapy, such as changing how antigens are expressed, manipulating immune checkpoints, and altering the tumor environment. These efforts aim to make cancer cells more recognizable by the immune system so they can be more easily attacked. Furthermore, advancements in personalized medicine have allowed for the creation of customized immunotherapies based on the unique characteristics of each patient’s tumor, enhancing the accuracy and effectiveness of these treatments. The TME is crucial in cancer progression and response to immunotherapy as it includes various cellular and molecular components that can support or suppress immune responses within the tumor. The goal is to convert a “cold tumor” (with limited immune activity) into a “hot tumor” (with strong immune infiltration) to improve treatment outcomes. Thus, several immunotherapy methods have been investigated to target the TME, including immune checkpoint inhibitors, cytokines, and adoptive T cell therapies. However, the challenges posed by TME heterogeneity remain substantial. Hence, researchers are exploring combination therapies that address multiple aspects of TME simultaneously. By disrupting immunosuppressive pathways and enhancing immune activation, these strategies aim to improve long-term treatment outcomes. Furthermore, personalized approaches that consider individual TME variations may boost the precision and effectiveness of cancer immunotherapy. Research findings indicate that T cells activated by immunotherapy can enhance lipid peroxidation specific to ferroptosis in cancer cells. This interaction highlights the potential synergy between immunotherapy and ferroptosis as a novel strategy for designing nanomedicine treatments.

### 3.2. Ferroptosis Triggers Immunogenic Cell Death, Dendritic Cell Maturation, and T Cell Activation

**Figure 4:**
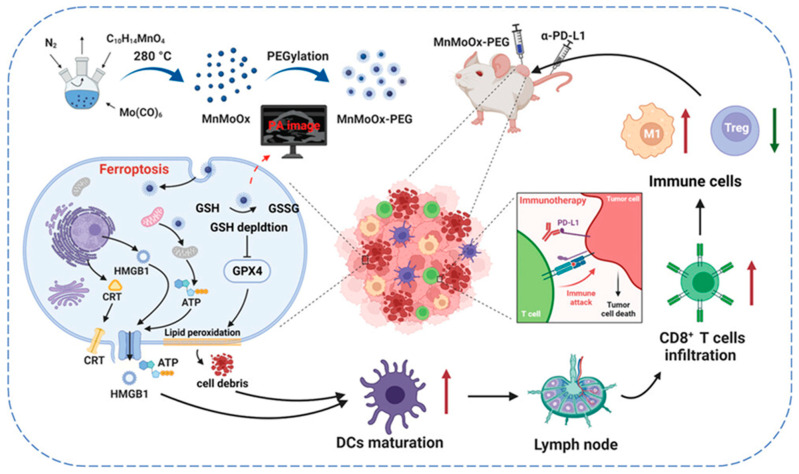
Role of inorganic nanomedicine (MnMoX) in ferroptosis-mediated cancer cell death, DAMP release, and induction of antitumor immunity. Red arrows indicate the process of ferroptosis leading to lipid peroxidation and cell death, while green arrows highlight the enhancement of immune responses, including M1 macrophage activation and CD8+ T cell infiltration, contributing to improved tumor immunotherapy. Reprinted from reference [48] with permission.

Damage-associated molecular patterns (DAMPs) are endogenous danger signals that cells release into the extracellular space in response to cellular damage or stress. When cancer is present, heightened tumor apoptosis induces stress and inflammatory signals, leading to DAMP constituents release and promoting immunogenic cell death (ICD) within cancer cells. The synergistic interaction between the secretion of DAMP constituents and ICD, particularly in response to some anticancer treatments, shows potential for attaining potent and efficacious antitumor immune responses. In the process of ferroptotic cell death, cancer cells release DAMPs such as high-mobility group box 1 (HMGB1), calreticulin (CRT), and adenosine triphosphate (ATP) into the TME. These DAMPs act as immunostimulant signals for recruiting and activating immune cells like dendritic cells (DCs) and macrophages, serving as cues that guide these immune cells to the site of dying tumor cells. Kim et al. developed hybrid core–shell vesicles (HCSVs) based on poly(lactic-co-glycolic acid) (PLGA), incorporating iron oxide nanocubes (IONCs) and ascorbic acid [46]. These HCSVs demonstrated a unique mechanism of action: when exposed to an exogenous magnetic field, they triggered the release of ascorbic acid. This promoted a redox reaction with the IONCs that converted ferric ions to ferrous ions and initiated the Fenton reaction. Notably, after 6 h of post-treatment with HCSVs and the magnetic field, CRT expression in TRAM-C1 cancer cells increased substantially. Furthermore, the increased release of DAMPs triggered the in vivo maturation of DCs at a rate of 27.9 ± 0.9% in HCSVs induced by ferroptosis. This value rose to 35.88 ± 1.8% in the HCSVs+MF group. By contrast, the control group had a maturation rate of 17 ± 6.3% [46]. The depletion of GSH during ferroptosis inactivated GPX4, causing uncontrolled lipid peroxidation. Zhao et al. developed a copper-based nanozyme system to enhance ferroptosis-induced ICD and trigger antitumor immune response [47]. Their copper nanozyme was structured with cholesterol oxidase and a lysyl oxidase inhibitor (Cu-BCO/CL) and induced mitochondrial dysfunction through ROS and GPXP depletion, leading to lipid peroxidation and ferroptosis. Therefore, 4T1 tumor cells treated with Cu-BCO/CL displayed increased CRT expression and HMGB1 release, heightening ICD. Additionally, CRT expression was elevated in Cu-BCO/CL-treated tumor tissues. The DC maturation rate rose from 17.4% in the control group to 26% with Cu-BCO treatment, promoting ferroptosis-mediated cell death. Combining Cu-BCO with CL further increased DC maturation to 43.8%. Furthermore, the treatment with Cu-BCO/CL resulted in a larger population of CD8+ and CD4+ T cells in the tumor tissue. Likewise, manganese molybdate nanoparticles (MnMoOx NPs) depleted intratumoral GSH levels and suppressed GPX4 expression, thereby initiating ferroptosis [48]. This process led to ICD and the subsequent release of DAMPs, enhancing tumor immunotherapy. The multivalent oxidation states of Mo (+5 and +6) and Mn (+3 and +4) within the MnMoOx NPs contributed to their robust GSH depletion capacity and efficient GPX4 inhibition [48] that resulted in ferroptosis and promoted ICD (shown in Figure 4). Notably, incubating MnMoOx NPs with CT26 cells resulted in ferroptosis-mediated toxicity, accompanied by increased CRT and HMGB1 release as well as a ten-fold elevation in ATP levels compared with the control group [48]. The intratumoral injection of MnMoOX provoked a notable upregulation of CRT and increased HMGB1 release to the ECM. The evaluation of DCs in tumor-draining lymph nodes (TDLNs) showed that intratumoral MnMoOX injections, administered three times, enhanced DC maturation from approximately 16.50% to 21.20%, boosting the local immune response against the tumor [48]. The MnMoOx injection led to an increase in CD8+ T cell infiltration into tumor tissue. In addition, inflammatory cytokines, including tumor necrosis factor-α (TNF-α), interleukin 6 (IL-6), and interleukin 12 p70 (IL-12p70), were upregulated in the lymph node [48].

### 3.3. Ferroptosis and Phototherapy

**Figure 5:**
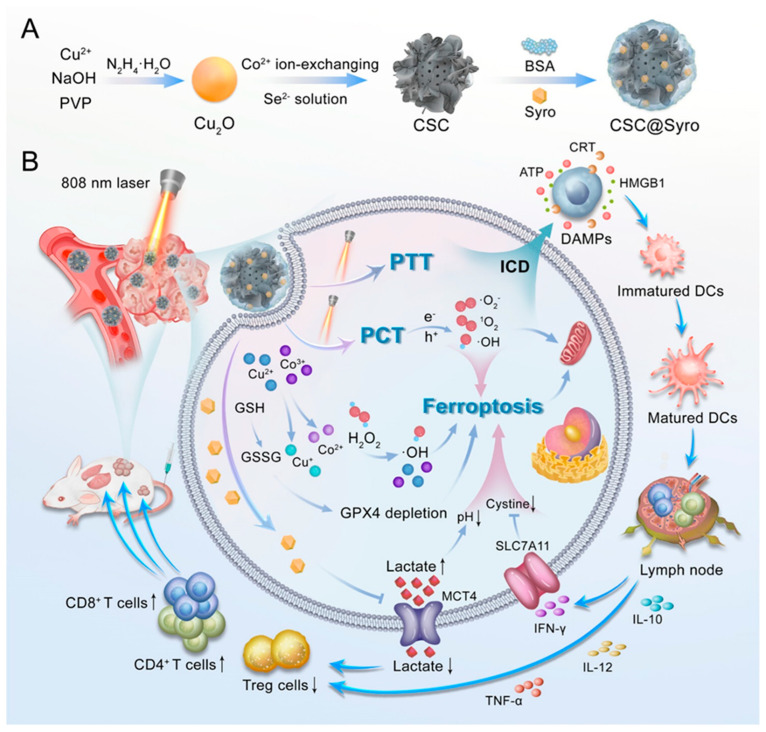
Integration of ferroptosis and photothermal therapy: Copper nanoparticles induce ferroptosis, leading to tumor cell death, DAMP release, and enhanced immune activation. In subfigure (**A**), CuSe/CoSe_2_ (CSC) nanoparticles are synthesized using Cu₂O and Co²⁺ ion-exchanging, followed by coating with BSA and Syro to form CSC@Syro. In subfigure (**B**), an 808 nm laser activates the nanoparticles, triggering photothermal therapy (PTT) and photocatalytic therapy (PCT), resulting in ferroptosis through GSH depletion and GPX4 inactivation. This process is illustrated with arrows indicating the flow of reactions from GSH depletion to lipid peroxidation and DAMP release, leading to dendritic cell maturation, CD8+ T cell infiltration, and an enhanced immune response. Reprinted from reference [49] with permission.

Photothermal materials absorb light, typically in the near-infrared (NIR) region, and convert it into heat, causing a localized temperature rise that destroys tumor cells through non-radiative mechanisms. Thus, combining ferroptosis with photothermal therapy creates a synergistic approach to destroying tumors and controlling their growth. As shown in Figure 5, Yang et al. developed novel nanoadjuvants using copper and cobalt (CSC@Syro). These heterostructural nanoadjuvants featured Cu^+^/Cu^2+^ and Co^2+^/Co^3+^ redox couples, which elevated endogenous hydrogen peroxide (H_2_O_2_) levels and promoted the generation of •OH during ferroptosis [49]. Both CSC and combined treatment with CSC@Syro substantially increased the expression of HMGB1 and CRT in 4T1 cells, compared with the control. Moreover, the ATP “find me” signal was four times stronger in the group treated with CSC@Syro and laser [49]. The heightened production of ROS during ferroptosis facilitated the abundant release of DAMPs, which promoted immune responses. The CSC treatment resulted in a DC maturation rate of 6.1%, whereas the CSC@Syro treatment achieved a maturation rate of 18.6%. By contrast, the control group exhibited a maturation rate of only 9.9%. Therefore, the increased DC maturation observed with both CSC and CSC@Syro treatments underscores the role of ferroptosis in promoting DAMP release and DC maturation [49]. Li et al. developed ultra-small MGNH Janus nanoparticles (MnFe_2_O_4_ @NaGdF4 @NLG919@HA) with an average particle size of 14.45 ± 2.38 nm for ferroptosis-activated combined photo-immunotherapy [50]. The MnFe_2_O_4_ in the MGNH nanoparticles reacted with endogenous H_2_O_2_ to promote lipid peroxidation in tumor cells and hampered GPX4 expression to induce ferroptosis. Ferroptotic cell death along with phototherapy-induced ICD, leads to DC maturation and activation of CD4+ and CD8+ T cells [50]. Overall, combining ferroptosis with photothermal therapy enhanced ICD and cancer immunotherapy.

### 3.4. Ferroptosis and Radiotherapy

**Figure 6:**
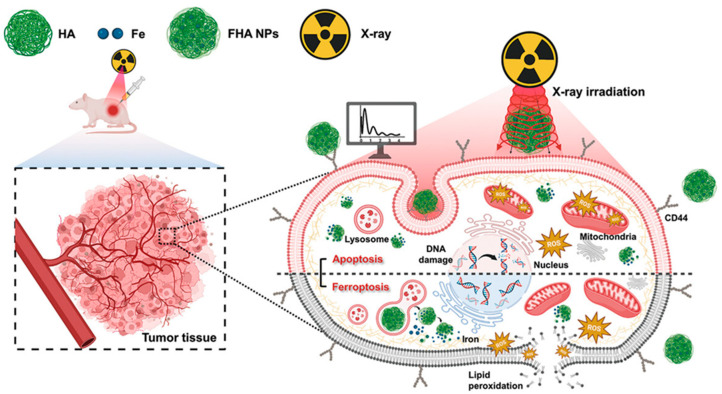
Mechanism of iron oxide nanoparticle-mediated ferroptosis combined with radiotherapy: overcoming radiation resistance and inducing tumor cell death. Reprinted from reference [51] with permission.

Radiotherapy is a common cancer treatment, but its effectiveness is often limited by radiation resistance. Thus, combining radiotherapy with ferroptosis has emerged as a promising strategy to enhance therapeutic effects as both ferroptosis and radiation-mediated apoptosis are induced simultaneously, avoiding radiation resistance mechanisms. As shown in Figure 6, Lee et al. developed hyaluronic acid-based iron oxide nanoparticles (FHA-NPs) to induce ferroptosis. Experiments and Monte Carlo simulations demonstrated an improved therapeutic effect [51]. The FHA-NP treatment resulted in higher ROS levels compared with X-ray treatment, with a notable increase observed during the combination therapy. Mouse model studies confirmed that combining ferroptosis with radiation therapy reduced the tumor volume. Accordingly, multiple doses of FHA-NPs, supplemented with radiation therapy, suppressed tumor growth. These findings highlight the synergistic role of radiation and ferroptosis in cancer therapy [51]. Moreover, the stability and enhanced cytotoxicity of FHA-NPs, combined with radiation treatment, has the potential to reduce common side effects associated with radiotherapy and pave the way for a safer X-ray therapy system [51]. Wang et al. reported that an iron oxide nanoprobe conjugated with an anti-PD-L1 antibody (Fe_3_O_4_-αPD-L1) targeted MDSCs and induced ferroptosis in these cells and, in turn, alleviated resistance to radiation therapy [52]. MDSC cells internalized the Fe_3_O_4_-αPD-L1 nanoprobes within 4 h. ROS levels were substantially higher in both the Fe_3_O_4_-αPD-L1 nanoprobes-only group and the radiation-only group compared with the control group, demonstrating the effects of the Fenton reaction and radiation-induced water radiolysis, respectively. Notably, the combination of Fe_3_O_4_-αPD-L1 nanoprobes with radiation resulted in the highest ROS production, with nearly 90% of MDSCs showing positive ROS signals [52]. In vivo antitumor studies in breast cancer and colorectal cancer models demonstrated that the combination of Fe_3_O_4_-αPD-L1 nanoprobes with radiation therapy suppressed tumor growth compared with controls and radiation therapy alone. These findings suggest that Fe_3_O_4_-αPD-L1 nanoprobes represent a promising targeted therapy to induce ferroptosis in MDSCs, potentially reshaping the immunosuppressive TME and enhancing radiation therapy efficacy [52].

### 3.5. Ferroptosis and Chemotherapy

**Figure 7:**
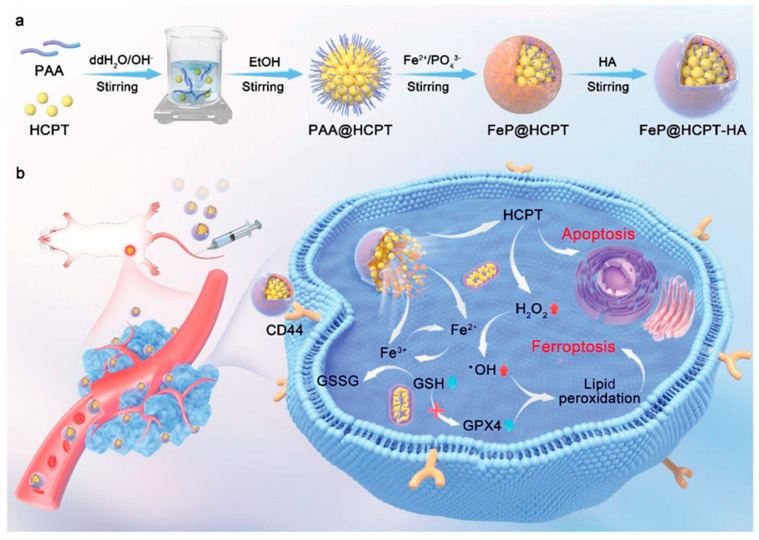
Inorganic nanoparticle-mediated ferroptosis through intracellular GSH depletion during chemotherapy to overcome chemoresistance. Subfigure (**a**) shows the synthesis process of FeP@HCPT-HA nanoparticles, starting with PAA and HCPT, followed by coating with iron phosphate (Fe²⁺/PO₄³⁻) and then hyaluronic acid (HA). Subfigure (**b**) illustrates the mechanism within cancer cells, where the nanoparticles target CD44 receptors, release HCPT, and induce apoptosis and ferroptosis. The red arrows highlight the increase in H₂O₂ and •OH production, driving ferroptosis, while the sky blue arrows show GSH depletion and GPX4 inactivation, leading to lipid peroxidation and cell death. Reprinted from reference [56] with permission.

Chemotherapy, a standard cancer treatment, often exhibits limited effectiveness because of its lack of specificity to tumor cells, leading to toxicity in healthy cells and drug resistance. The elevated levels of GSH in cancer cells are a major contributor to this resistance [53]. GSH binds to drugs, interacts with ROS, prevents damage to proteins or DNA, and participates in DNA repair. GSH levels are exceptionally high in cancer cells to combat oxidative stress from ROS and reactive nitrogen species (RNS), byproducts of redox reactions and cellular respiration. The elevated GSH levels aid in the survival and rapid proliferation of cancer cells, reducing the effectiveness of anticancer drugs by neutralizing ROS/RNS. Hence, depleting GSH is crucial to improve the efficacy of anticancer agents. Using inorganic nanoparticles to induce ferroptosis, a type of cell death that depletes intracellular GSH, is thus a promising strategy. This approach effectively targets cancer cells and overcomes the chemoresistance caused by excess GSH. Thus, the therapeutic potential against cancer can be enhanced by combining chemotherapy with nanoparticle-mediated ferroptosis [54,55]. Cai et al. designed biodegradable ferric phosphate nanoparticles combined with the chemotherapeutic drug camptothecin (FeP@HCPT-HA) to deplete GSH and promote chemo-ferroptosis [56]. FeP@HCPT-HA released HCPT and ferric ions (Fe^2+^, Fe^3+^) in the acidic tumor environment. Furthermore, HCPT induced apoptosis and generated excess H_2_O_2_, aiding Fe^2+^-mediated ferroptosis. The released Fe^3+^ reacted with GSH, hampering GPX4 expression via a redox reaction (Figure 7). Hence, the combination of chemotherapy and ferroptosis suppresses tumor growth more effectively than single therapeutic methods [56].

## 4. Clinical Challenges and Opportunities in Inorganic Nanoparticle-Based Ferroptosis

The combination of inorganic-based ferroptosis-inducing NPs and immunotherapy holds immense promise for cancer treatment, but several key challenges must be addressed before clinical translation and industrialization can become a reality. One of the primary issues is ensuring the safety and selectivity of inorganic NPs. While these NPs are effective in inducing ferroptosis, the potential for off-target effects, such as toxicity to healthy cells and long-term accumulation in organs, raises concerns. The metal-based nature of these NPs could lead to excessive ROS production, which, while effective against tumor cells, may also cause damage to surrounding tissues. Therefore, developing nanoparticles with improved specificity and biocompatibility will be crucial for reducing side effects and ensuring patient safety.

Another significant barrier is the scalability and reproducibility of manufacturing these complex NP systems. Industrializing the production of highly specialized inorganic NPs requires stringent control over size, composition, and surface modifications to ensure consistent therapeutic efficacy. Current NP synthesis methods may not be fully scalable, and any variability in production could lead to inconsistencies in treatment outcomes. Therefore, advancements in large-scale manufacturing techniques will be essential to bringing these therapies from the lab to the clinic.

From a clinical perspective, more robust preclinical and clinical trials are needed to fully understand the interactions between ferroptosis and the immune system in diverse tumor environments. The TME is highly variable across different cancer types, and how this affects the efficacy of ferroptosis and immunotherapy combinations is not yet fully understood. For instance, the immunosuppressive TME in some cancers may limit the effectiveness of immunotherapy, even when combined with ferroptosis induction. Furthermore, the mechanisms by which ferroptosis influences immune cell recruitment and activation need further exploration to optimize the synergy between these treatments. Regulatory hurdles also pose a challenge as the approval process for nanoparticle-based therapies is often more stringent due to concerns about long-term toxicity, biodistribution, and environmental impact. Comprehensive toxicological studies and long-term safety data will be required to satisfy regulatory bodies, potentially slowing down the approval process for these innovative treatments.

In summary, while the combination of inorganic-based ferroptosis and immunotherapy holds transformative potential for cancer treatment, its path to clinical use and industrial-scale production is fraught with challenges. Addressing safety concerns, improving manufacturing scalability, understanding tumor-specific responses, and navigating regulatory frameworks will be critical steps toward realizing the full potential of this therapeutic strategy.

## 5. Conclusions and Future Perspectives

The global incidence of cancer is rising, and traditional treatments such as chemotherapy and radiotherapy often result in side effects and drug resistance. On the other hand, emerging treatments like gene therapy and immunotherapy show promise but require more research to determine their safety and long-term effects. Ferroptosis, a regulated cell death mechanism identified in 2012, offers a novel approach to cancer therapy by leveraging the Fenton reaction and inhibiting GPX4. However, metabolic adaptation by tumor cells and the rapid elimination of ferroptosis-inducing small molecules in the bloodstream limit this process’ effectiveness. Thus, the enhanced efficacy and specificity of these therapies necessitate further research.

Nanotechnology has highlighted the potential of ferroptosis-inducing inorganic nanomedicines, which enhance drug stability, safety, targeting, and controlled release. These nanomedicines offer various advantages, such as passive targeting of tumors through the enhanced permeability and retention (EPR) effect and active targeting by modifying nanoparticles with antibodies specific to tumor surfaces. Furthermore, the large hydrodynamic size reduces renal clearance and prolongs their half-life in the bloodstream. Thus, combining ferroptosis-inducing inorganic nanomedicines with chemotherapy, phototherapy, and immunotherapy can result in more effective treatments through synergistic effects. These advances make ferroptosis-inducing inorganic nanomedicines a promising approach to cancer therapy. Nonetheless, several challenges remain. The limited intracellular Fenton reaction, which results in low ROS levels in weakly acidic environments, is a major problem. Thus, understanding the relationship between ferroptosis, immunity, and the TME is crucial for developing targeted inorganic nanomaterials. Strategies for guiding nanomaterial targeting in patients and overcoming tumor resistance also need further exploration. Furthermore, the mechanisms of immunotherapy and its combination with ferroptosis require more research to avoid potential side effects. While ferroptosis-inducing nanoparticles offer notable advantages, the potential risks associated with metal nanoparticles and excessive ROS generation must be considered. These risks include toxicity to adjacent normal cells and unknown long-term health effects; thus, thorough in vivo evaluations are required to ensure safety and efficacy. In addition, nanoparticles should be developed with high selectivity for cancer cells to minimize unintended consequences. Hence, interdisciplinary research is essential to designing effective and safe cancer therapies.

## Abbreviations

ROS	Reactive Oxygen Species
L-ROS	Lipid Reactive Oxygen Species
PUFAs	Polyunsaturated Fatty Acids
GPX4	Glutathione Peroxidase 4
GSH	Glutathione
HSBP1	Heat Shock Factor-Binding Protein 1
Nrf2	Nuclear Factor Erythroid 2-Related Factor 2
P53	Tumor Protein P53
ACSL4	Acyl-CoA Synthetase Long-Chain Family Member 4
MUFAs	Monounsaturated Fatty Acids
NPs	Nanoparticles
XC—	Cystine/Glutamate Antiporter System
RSL3	Ras-Selective Lethal 3
FIN56	Ferroptosis Inducing Compound 56
DPI2	Diphenyleneiodonium Chloride 2
Fe	Iron
Mn	Manganese
Cu	Copper
Pt	Platinum
PTT	Photothermal Therapy
PDT	Photodynamic Therapy
PEG	Poly(ethylene) Glycol
MRI	Magnetic Resonance Imaging
IFN-γ	Interferon Gamma
NOXs	NADPH Oxidases
TA	Tannic Acid
1O2	Singlet Oxygen
OH	Hydroxyl Radicals
TME	Tumor Microenvironment
Cu	Copper
Cin	Cinnamaldehyde
GOx	Glucose Oxidase
Au	Gold
TAX1BP1	Tax1 Binding Protein 1
Mn	Manganese
Pt (IV)	Platinum (IV)
ZAL	Zalcitabine
BSA	Bovine Serum Albumin
DAMPs	Damage-Associated Molecular Patterns
ICD	Immunogenic Cell Death
HMGB1	High-Mobility Group Box 1
CRT	Calreticulin
ATP	Adenosine Triphosphate
TME	Tumor Microenvironment
DCs	Dendritic Cells
MDSCs	Myeloid-derived suppressor cells
PLGA	Poly(lactic-co-glycolic Acid)
TNF-α	Tumor Necrosis Factor Alpha
IL-6	Interleukin 6
IL-12p70	Interleukin 12 p70
RNS	Reactive Nitrogen Species
